# In-silico prediction of novel genes responsive to drought and salinity stress tolerance in bread wheat (*Triticum aestivum*)

**DOI:** 10.1371/journal.pone.0223962

**Published:** 2019-10-31

**Authors:** Laila Dabab Nahas, Naim Al-Husein, Ghinwa Lababidi, Aladdin Hamwieh

**Affiliations:** 1 Biotechnology Engineering Dept/Technological Engineering Faculty/University of Aleppo, Aleppo, Syria; 2 General Commission for Scientific Agricultural Research (GCSAR)/Ministry of Agriculture, Aleppo, Syria; 3 International Center for Agricultural Research in the Dry Areas (ICARDA), Cairo, Egypt; Saint Mary’s University, CANADA

## Abstract

Common wheat (*Triticum aestivum*) is the most widely grown cereal crop and is cultivated extensively in dry regions. Water shortage, resulting from either drought or salinity, leads to slow growth and loss of wheat yield. In order to predict new genes responsive to the drought and salt stresses in wheat, 6,717 expressed sequence tags (ESTs), expressed in drought and salinity stress conditions were collected from the National Center for Biotechnology Information (NCBI). The downloaded ESTs were clustered and assembled into 354 contigs; 14 transcription factor families in 29 contigs were identified. In addition, 119 contigs were organized in five enzyme classes. Biological functions were obtained for only 324 of the 354 contigs using gene ontology. In addition, using Kyoto Encyclopedia of Genes and Genomes database, 191 metabolic pathways were identified. The remaining contigs were used for further analysis and the search for new genes responsive to drought and salt stresses. These contigs were mapped on the International Wheat Genome Sequencing Consortium RefSeq v1.0 assembly, the most complete version of the reference sequence of the bread wheat variety Chinese Spring. They were found to have from one to three locations on the subgenomes A, B, and D. Full-length gene sequences were designed for these contigs, which were further validated using promoter analysis. These predicted genes may have applications in molecular breeding programs and wheat drought and salinity research.

## Introduction

Bread wheat (*Triticum aestivum*) is an important staple food around the world. It resulted from hybridization between cultivated tetraploid wheat (AABB, *T. dicoccoides*) and diploid goatgrass (DD, *Aegilops tauschii*) about 8,000 years ago [1]. Despite both the fundamental knowledge gained from relevant studies concerning the wheat genome and the importance of the crop, a comprehensive genome-wide analysis of gene content was not conducted until recently. This was because of the large size, repeat content, and polyploid complexity of the genome [2]. However, assembly of the 17-Gb allohexaploid genome of *T. aestivum* faced major difficulties, because it is composed of three large, repetitive, and closely related genomes [3]. In addition, the genome is formed of nearly 80% repeats, primarily retro-elements, with a gene density ranging between 1/87 and 1/184 kb [4]. Improved versions of wheat assembly were released in 2016 and 2017. However, use of these data was restricted until the International Wheat Genome Sequencing Consortium (IWGSC) published its analysis (https://www.wheatgenome.org/). On 17 August 2018, the IWGSC RefSeq v1.0 assembly, the first most complete version of the reference sequence of the bread wheat variety Chinese Spring, was made publicly available without restriction at Unité de Recherche Génomique Info [5]. This advance will lead to new discoveries of novel genes and an understanding of how genes interact, are activated and suppressed across divergent tissues, and of the development stages and environmental stresses. Subsequently, such new discoveries will result in wheat improvement [6]. Efforts have been made to develop drought- and salt-tolerant varieties [7]. Drought and salinity stresses show high degrees of similarity in a number of different ways, also in their molecular and genetic effects [8, 9]. These similarities include metabolic processes, such as increases in levels of plant hormonal processes (e.g. of abscisic acid, ABA) or decreases in photosynthesis. Additionally, high intracellular concentrations of sodium and chloride ions because of drought stress lead to increased salinity stress [10]. In a concept known as cross-tolerance, plants use common pathways and components in response to stresses. These allow them to adjust to a range of divergent stresses after being exposed to one specific stress [11, 12]. Therefore, a drought-tolerant species can also be salinity tolerant and vice-versa, and possess similar mechanisms to interact with these stresses [13]. Many elements play major roles in response to abiotic stresses, including transcription factors (TFs), which regulate gene expression. There is a focus on TFs for the genetic engineering of stress tolerance, which has resulted in a wide array of stress response genes that are up- or down-regulated by overexpression of a single TF with implications for various stress pathways [7]. Known examples of TFs with important roles in dehydration and salinity include abscisic acid-responsive element-binding protein (AREB)/ABFs (ABA responsive agent binding factors) function in ABA-dependent gene expression, and dehydration-responsive element-binding protein1 (DREB1)/CBF and DREB2 function in ABA-independent gene expression. MYB (myeloblastosis oncogenes)/MYC (v-myc avian myelocytomatosis viral oncogene homolog) also have an essential role in abiotic stresses, like salinity, cold, and drought [7, 14]. Sequencing projects for ESTs have been performed for various organisms, creating millions of short, single-pass nucleotide-sequence reads, and are available from EST databases. Inclusive computational strategies have been advanced to regulate and analyze EST data for gene discovery, transcription, and functional annotation for products of a putative gene [15]. Similar studies on EST sequences have been performed on wheat for biotic stress and on Brassica rapa [16]. In the present study, TFs and enzymes were initially identified. Then, functional characterization was performed to identify their role in drought- and salt-stress mechanisms, since both salt and drought finally result in dehydration and osmotic imbalance of the cell. As there are a lot of components common for both, stresses cross-talk with each other on cooperative pathways to tolerant the stress. Functional characterization was done using the basic local alignment search tool (BLAST). These were then grouped into categories using the gene ontology (GO) vocabulary. We used BLAT (a BLAST-like alignment tool) to determine the positions of candidate sequences on the wheat genome. BLAT is a sequence alignment tool similar to BLAST but it is structured differently. It quickly finds similarities in DNA and proteins but requires an exact or nearly exact match to find a hit [17]. Gene prediction was made using the online tool FGENESH. Putative genes were further validated in-silico, based on examination of cis-regulatory elements in the promoter region. The purpose of promoter analysis is to determine the potential sequences of cis-acting DNA that may be controlling the candidate gene expression. In plants, the transcriptional regulation is mediated by the binding of TFs to specific cis-acting regulatory elements (CAREs) on DNA and begins the directness of transcription. The CAREs are short, conserved DNA motifs of 5-20 nucleotides, mostly found in the promoter region of a gene for the specific binding of RNA polymerase and for dynamic transcription in specific tissues at specific times. To organize and control gene expression specifically, TFs interact with these specific DNA elements, other TFs, and the major transcriptional machinery [18]. In addition, enzymatic classes give us a good picture of the reactions that happen during these stresses. In our study, first we had a general view on the EST-contigs data. Second, we searched for new genes and confirmed our results with some tools and analysis. The genes identified will be novel potential genes in the co-network of drought and salt resistance mechanisms in wheat. Similar work has been done for sorghum, maize, and rice [19–21]. The findings will be useful in developing wheat varieties resistant to both drought and salinity stress.

## Materials and methods

### Retrieving the data, cleaning, and assemblage

There were 6,717 ESTs of *T. aestivum* obtained from the EST database in NCBI (www.ncbi.nlm.nih.gov) containing all the entries from the GenBank database of the EST or cDNA categories that have been expressed in drought and saline conditions until now. The ESTs were masked to exclude sequence parts that could cause incorrect clustering [15] and then were masked for genomic repeats, vector sequence, low complexity sequence (including poly-A tails), and sequencing artifacts by EGassembler [22]. The processed EST sequences were grouped into clusters using the CAP3 program [23]. Sequences that could not be grouped, because of low similarity with other ESTs, resulted in singletons.

### Identification of TFs and enzymes

All assembled contigs were analyzed using the PlantTFcat online tool (http://plantgrn.noble.org/PlantTFcat) for TFs and the KEGG database resource (https://www.genome.jp/kegg/) for enzymes.

### Functional analysis of EST-contigs

Functional analysis of the EST-contigs was performed using Blast2GO v 2.5, which is a gene ontology-based annotation tool that is effective in functional characterization of plant sequence data [24]. The assembled EST-contigs were first translated in all reading frames and then compared with the NCBI nr protein sequence database to identify potential translation products using BLASTX. All sequences of EST-contigs homologous with annotated proteins in the NCBI nr database were selected for functional characterization. The EST-contig sequences were then categorized into three groups according to the GO vocabulary: molecular function, biological process, and cellular component. Remaining EST-contigs that had no BLAST hit and GO prediction were further analyzed for identification of novel candidate genes related to drought and salt stresses in wheat. In addition, KEGG pathways were detected to the contigs assembled sequences using the online KEGG Automatic Annotation Server (KAAS) 60, (http://www.genome.jp/kegg/kaas). The single-directional best hit (SBH) method was used in KEGG analysis.

### Gene identification using EST-contigs

The EST-contigs, for which no BLAST hit and GO terms were assigned, were aligned on the comprehensive and correctness reference sequence of the allohexaploid wheat IWGSC RefSeqv1.0 assembly, using stand-alone BLAT with 97% similarity [17]. The length of the aligned EST-contigs on the wheat genome was further extended by 1 kb upstream and downstream—these sequences were used to predict the structure of genes with transcription start site (TSS), poly-A tails at the extremes, and coding sequences (CDS) between, using the FGENESH gene prediction program [25]. The EST-contigs for predicted genes with no BLAST hits were translated in all reading frames again and compared with the InterPro protein sequence databases (https://www.ebi.ac.uk/interpro/about.html) to identify potential functions using BLASTX.

### Promoter analysis of candidate genes

An in-silico analysis was used to validate the above candidate genes by examining the promoter region of the predicted genes for the cis-regulatory elements. The cis-regulatory elements in the promoter regions were obtained from different resources and published literature, and then examined using the PlantPAN website (http://PlantPAN2.itps.ncku.edu.tw). PlantPAN provides resources for detecting transcription factor binding sites (TFBSs), corresponding TFs, CpG islands, and tandem repeats in plant promoters [26].

## Results

### Assembling of ESTs into contigs

A total of 6,717 EST sequences, related to drought- and salt-stress tolerance of wheat extracted from roots and leaves, were downloaded from GeneBank. The average length of these ESTs was 650 bp and primary sequence analysis showed a total length of 4,365,910 bp and a total GC count of the non-redundant EST collection was 55% Of the 6,717 EST sequences (Table 1), in the first step of EGassembler, it screened EST sequences for repeats and low complexity sequences. Therefore, the total elements were masked and trimmed 2,600 bp, i.e. about 0.06% of the total size of the query sequence. Then using another step from the same program, the remaining EST sequences for *T. aestivum* were assembled into 354 contigs and 5,869 singletons. Most of the contigs consisted of two or three ESTs. These assembled ESTs accounted for only 12.62% of all ESTs. Singletons representing slightly expressed transcripts could not be assembled into larger contigs. These singletons may represent expressed genes for which only single mRNA was collected or may result from contamination and were not considered for further analysis.

**Table 1:** Summary of EST analysis conducted with EGassembler.

Feature	Numbers
*TotalnumberofESTs*	6,717
*ESTtotalnucleotides*(*nt*)	4,366,252
*Clusterscount*	6,223
*Singleton*	5,869
*Contig*	354
*AverageESTlength*(*nt*)	650
*AverageGCcontent*(%)	55

### Identification of TFs and enzymes

**Fig 1:**
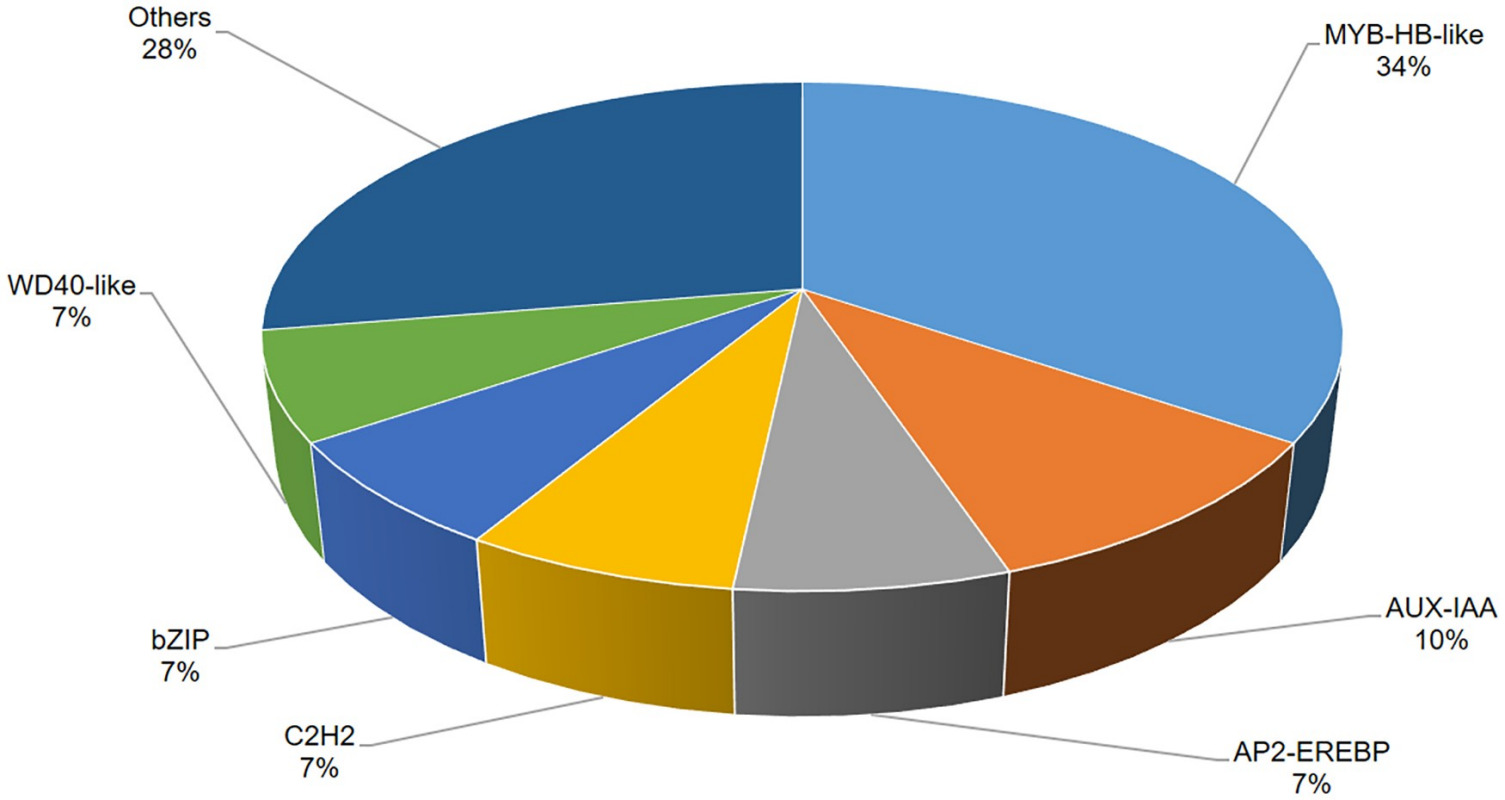
Distribution of TFs in the EST-contigs.

**Fig 2:**
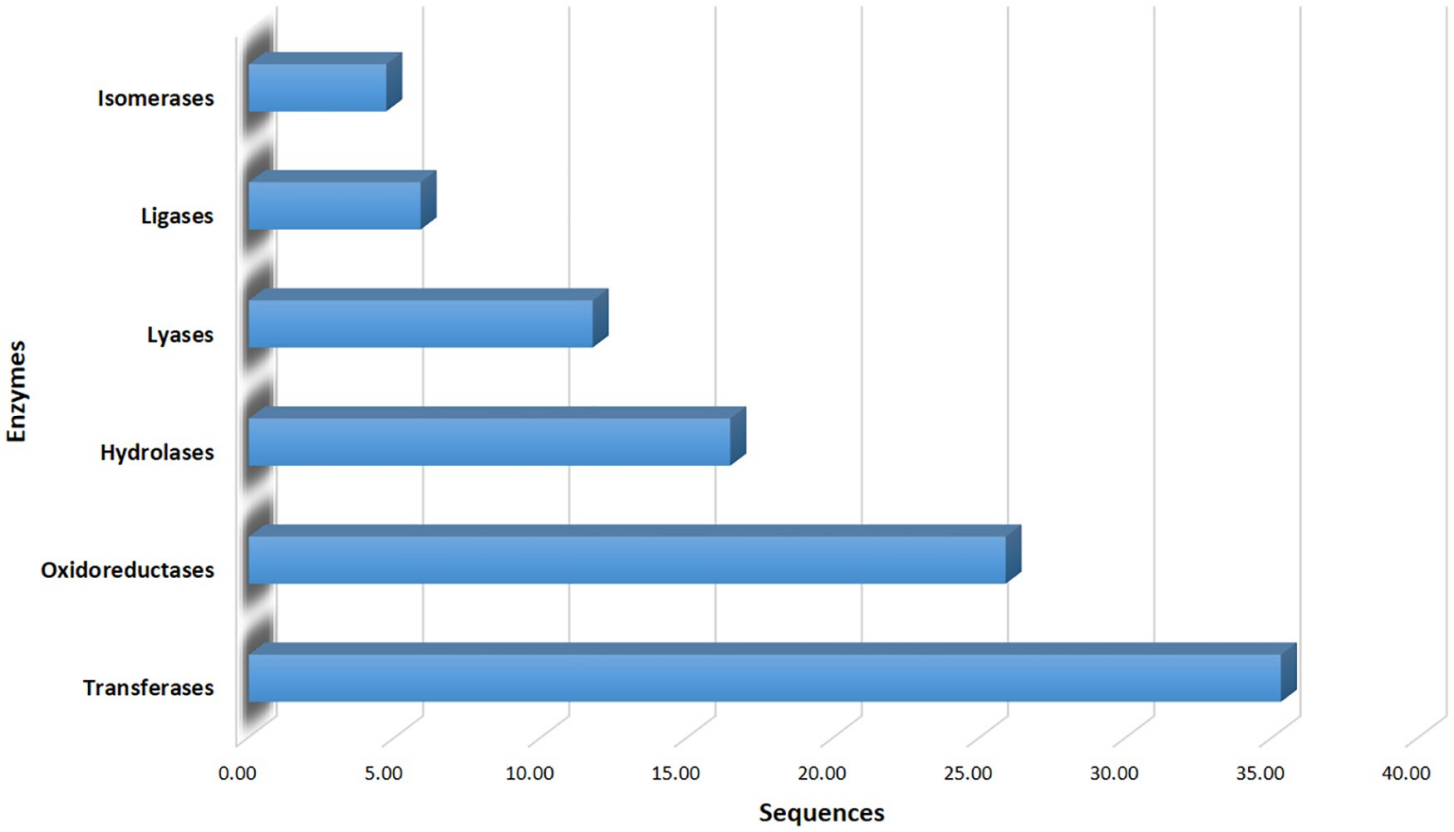
Distribution of enzymes in the EST-contigs.

Twenty-nine contigs were identified and sorted into 14 putative TF families (S1 Table). Among the 14 TF families (Fig 1), MYB-HB-like is the most abundant category (34%), followed at 10% by AUX-IAA, and 7% each for AP2-EREBP, WD40-like, bZIP, and C2H2 TFs. In addition, 119 contigs recognized and represented 85 sub enzymes (S2 Table) classified into the main six enzyme classes (Fig 2). The three majors are transferases which is the biggest set (35%), followed by oxidoreductases (26%), and hydrolases (16%).

### Functional annotation of EST-contigs

**Fig 3:**
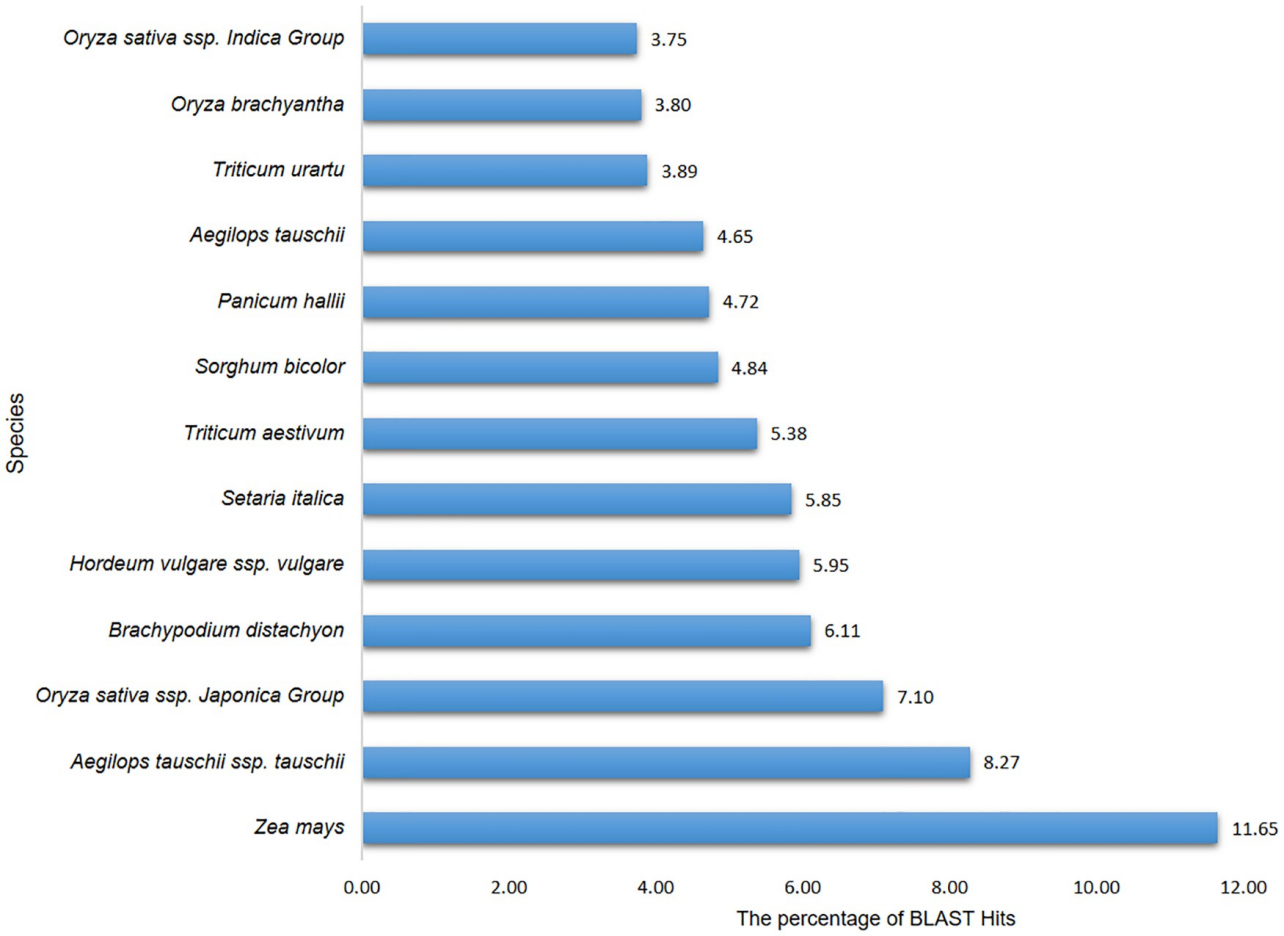
Species distribution of *T. aestivum* EST-contigs.

**Fig 4:**
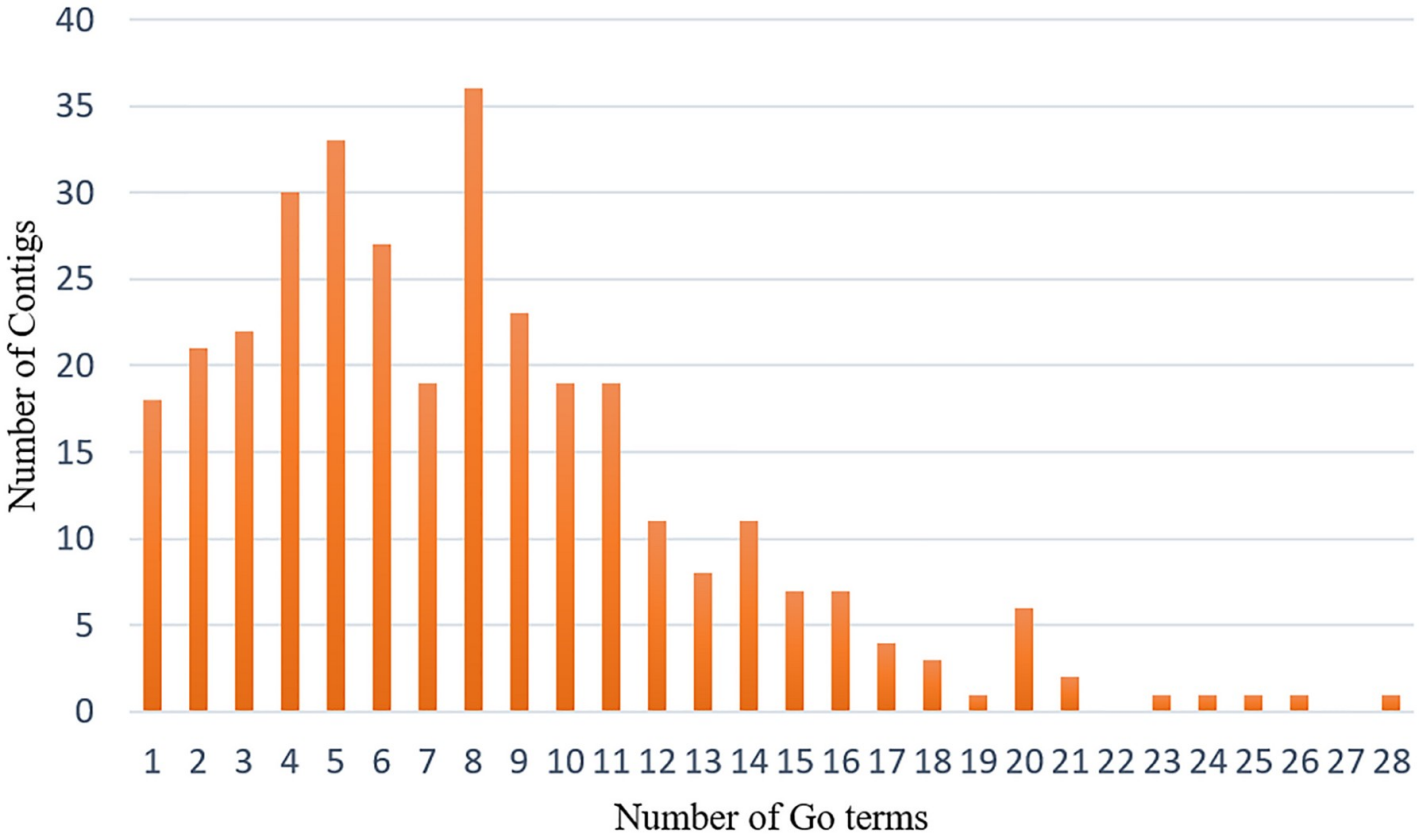
Distribution of numbers of EST-contigs vs. numbers of GO terms.

**Fig 5:**
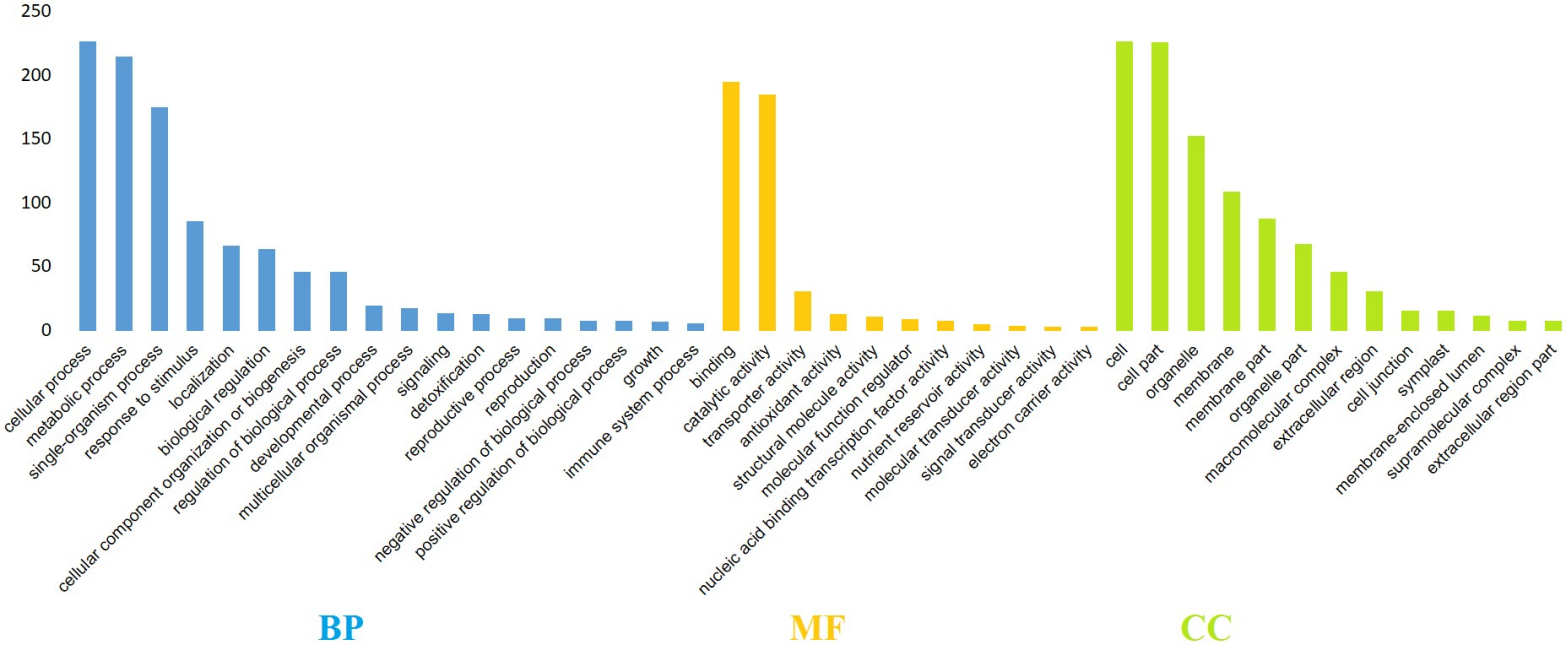
Gene ontology classification of EST-contig sequences. Biological process (BP), molecular function (MF), and cellular component (CC).

In order to functionally characterize the 354 assembled and translated EST-contigs, they were compared with the NCBI nr database and thus 349 were selected. BLAST results showed that more than 5.38% of these EST-contigs were exclusive to *T. aestivum* and more than 50% were conserved across higher plant species *Zea mays*, *Aegilops tauschii*, *Oryza sativa*, *Brachypodium distachyon*, *Hordeum vulgare*, and *Sorghum bicolor*. The percentage of BLAST hits for *Z. mays*, *A. tauschii*, and *O. sativa* was 11.65%, 8.27%, and 7.1%, respectively (Fig 3). These 349 EST-contigs were then subjected to GO functional classification; however, GO terms were available for only 324 EST-contigs. There were 1,738 GO terms retrieved, indicating an average of five GO terms per contig; there was a maximum of 28 GO terms for one contig and a minimum of one GO term for 18 contigs (Fig 4). The EST-contig sequences were grouped according to the GO vocabulary of molecular function, cellular component, and biological process at Level 2 (Fig 5). The 1,122 KEGG annotated contigs were categorized into six different functional groups (S3 Table). Of these, 235 contigs were classified into the “metabolism”, with most of them involved in “carbohydrate metabolism” (23.4%), “energy metabolism” (17.87%), “amino acid metabolism” (16.17%), “biosynthesis of other secondary metabolites” (11.06%), “lipid metabolism” (9.42%) and other sub-categories. This suggests that carbohydrate, amino acids and energy metabolism were active under salt and drought stresses. In addition, Cellular processes were represented by 62 contigs consisting of “transport and catabolism” (53.22%), “cell growth and death” (33.87%) and “cell communication” (12.9%). In addition to Cellular processes, sequences were also classified into the “genetic information processing, which accounted for 45 contigs of the KEGG annotated sequences, most of them were involved in “Folding, sorting and degradation” (53.33%), followed by “Translation” (22.22%) and “Transcription” (17.78). Additionally, 38 contigs were classified into “environmental information processing” including “signal transduction”.

### Candidate gene prediction from EST-contigs

**Fig 6:**
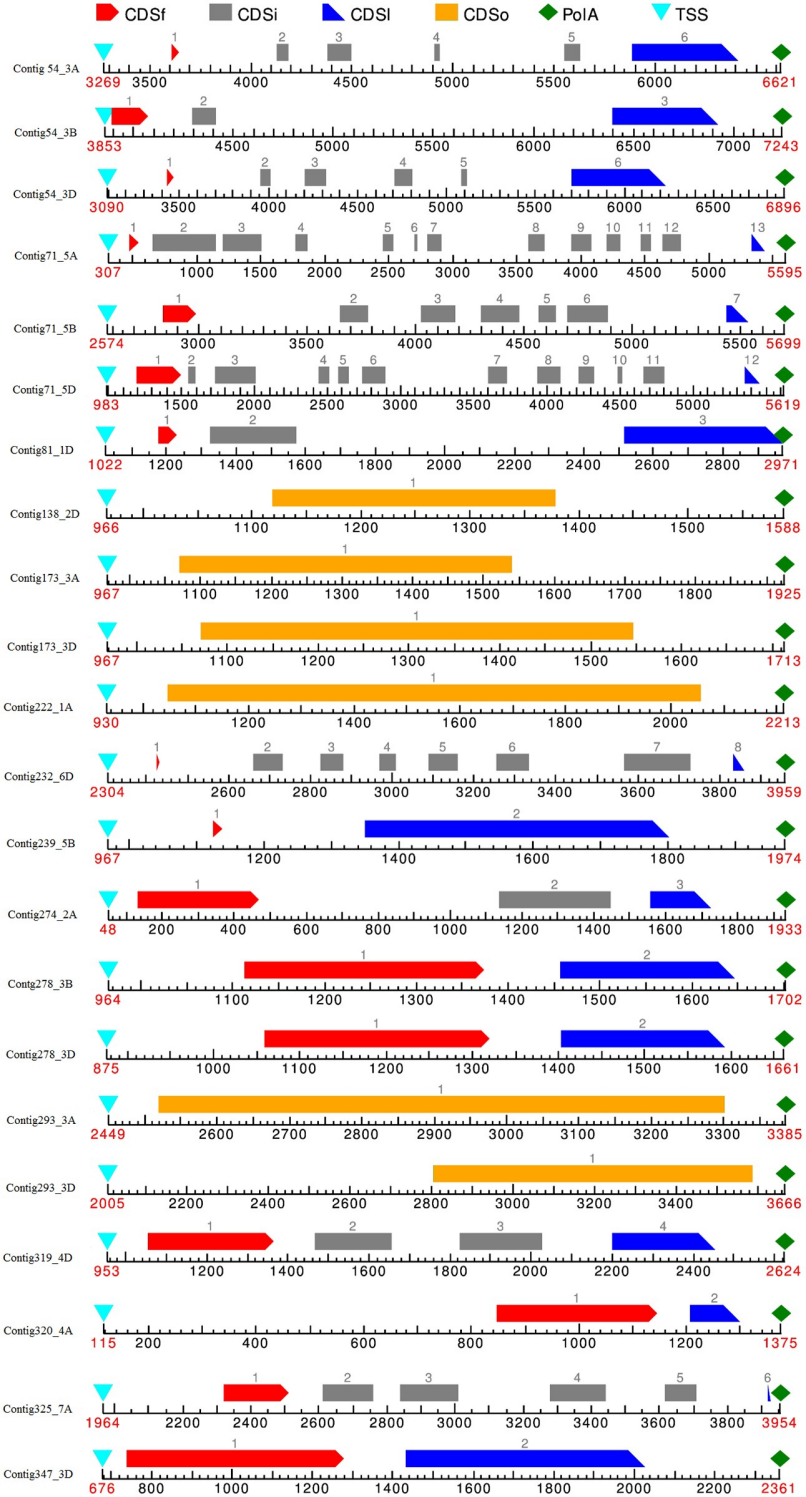
Gene structures of the production genes. CD (CDSf {First Coding segment}, CDSi {Internal Coding segment}, CDSl {Last Coding segment}, CDSo {one Coding segment}).

Of the 354 EST-contigs, the 30 with no BLASTX hit and GO terms found were considered for candidate gene prediction of drought- and salt-stress tolerant genes. These EST-contigs were aligned against the *T. aestivum* genome using BLAT, and five could not be aligned. The remaining 25 contigs that were aligned with different score ranges were further extended to 1 kb upstream and downstream on the genome, and this resulted in 15 contigs with one to three locations on the three subgenomes. In total, 22 such genomic regions—with TSS, poly-A tails at the extremes, and CDS between—were obtained as novel candidate genes out of 15 contigs (Fig 6 and S4 Table). These novel candidate genes were distributed among two-thirds of the chromosomes, including 1A, 1D, 2A, 2D, 3A, 3B, 3D, 4A, 4D, 5A, 5B, 5D, 6D, and 7A, each with one, two, or three loci (Table 2).

**Table 2:** Coding positions of the in-silico validated candidate genes.

Number	Contig ID	Chromosome No	Gene ID	Start (bp)	End (bp)
1	contig54	3A	TraesCS3A02G167700	174283376	174288611
2	contig54	3B	TraesCS3B02G198700	226085266	226091014
3	contig54	3D	TraesCS3D02G173800	155555503	155560557
4	contig71	5A	TraesCS5A02G271700	481910442	481914111
5	contig71	5B	TraesCS5B02G272000	457343326	457347105
6	contig71	5D	TraesCS5D02G279400	381410986	381414680
7	contig85	1D	TraesCS1D02G102400	90640055	90641897
8	contig138	2D	TraesCS2D02G533600	617995074	617995688
9	contig173	3A	TraesCS3A02G148700	131458348	131459078
1	contig173	3D	TraesCS3D02G156500	123908560	123909337
11	contig222	1A	TraesCS1A02G012600	7185452	7186567
12	contig232	6D	TraesCS6D01G065900	32296873	32299543
13	contig239	5B	*-	445447116	445448056
14	contig274	2A	TraesCS2A02G127100	75134856	75135248
15	contig278	3B	TraesCS3B02G409300	644910278	644910863
16	contig278	3D	TraesCS3D02G369800	482595907	482596438
17	contig293	3A	TraesCS3A02G024900	13354484	13356489
18	contig293	3D	TraesCS3D02G021200	7026349	7028651
19	contig319	4D	TraesCS4D02G022300	9390993	9391988
20	contig320	4A	*-	78024571	78025890
21	contig325	7A	*-	271633990	271636634
22	contig347	3D	TraesCS3D02G540600	611918534	611919445

*—means no predicted genes found in the IWGSC RefSeqv1.0 assembly.

### Promoter analysis of novel candidate genes

**Fig 7:**
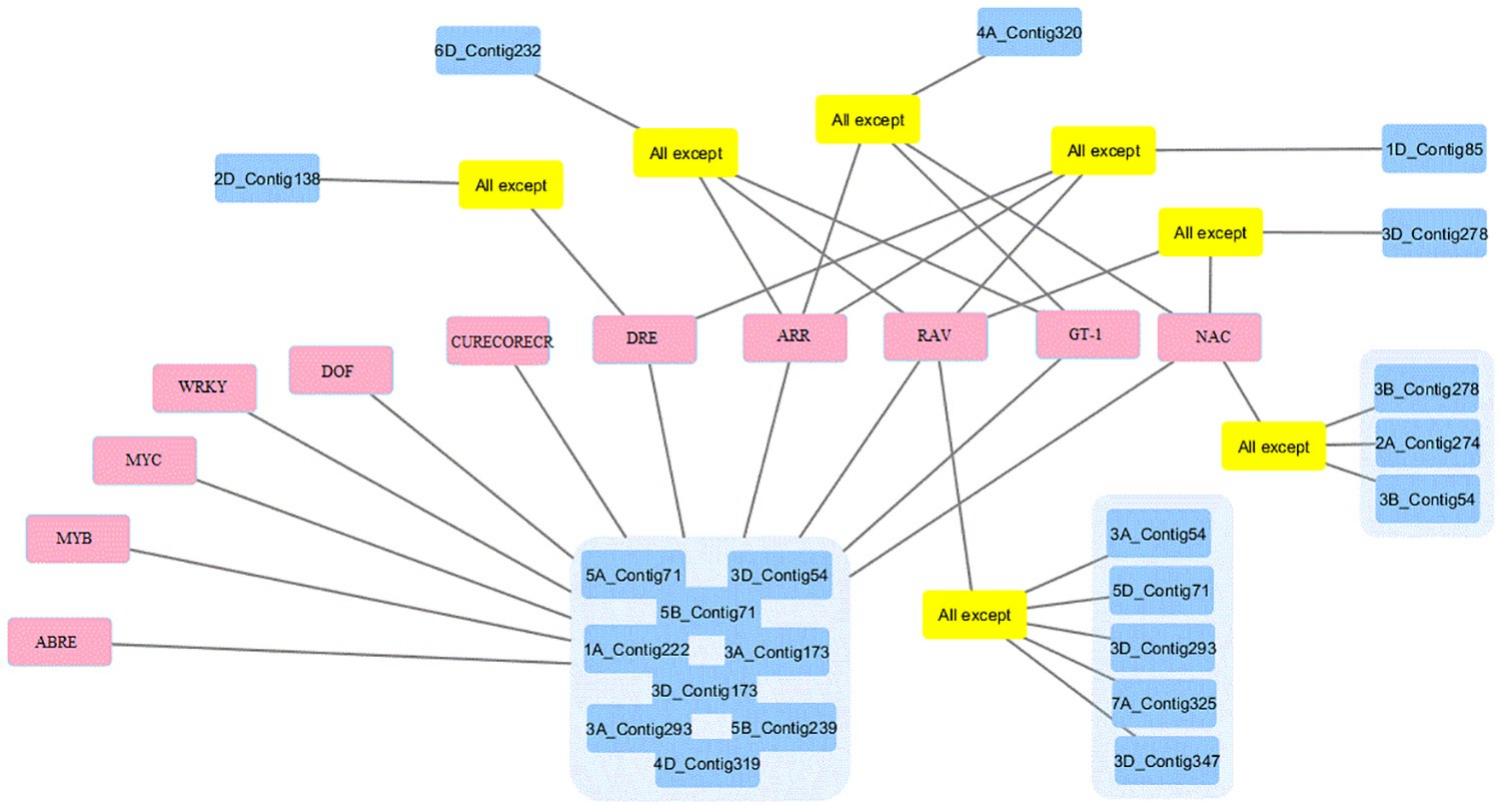
Presence of salt and drought stresses cis-elements in the candidate genes. The pink rectangles represent the cis-elements. Blue rectangles represent the expected genes in this study. They contain the chromosome homology number and name of the contig expected to found there. ‘All except’ yellow rectangles mean that the gene contains all 10 cis-elements except those linked with them.

The collected drought and salt-stress responsive cis-elements of ABRE, DREs, MYB, MYC, WRKY, ARR, DOF, RAV, GT-1, CURECORECR, and NAC, along with their conserved cis motif sequences, are given in (Table 3). The S5 Table provides a list of all reported drought- and salt-responsive cis-elements present in the candidate genes. More than one-third of the expected genes (nine expected genes, numbers 3, 4, 5, 9, 10, 11, 13, 17, and 19) had the entire set of reported drought and salt-stress responsive cis-elements. Another nine of them (expected genes, numbers 1, 2, 6, 8, 14, 15, 18, 21, and 22) had 10. While one (expected gene, number 16) had nine and three of them (expected genes, numbers 7, 12, and 20) had eight cis-elements present in the promoter region (Fig 7). The cis-elements were located and their positions mapped starting from the TSS to the 1-kb upstream region.

**Table 3:** Table Cis-element and conserved cis motif sequences related to the expression of drought and salt-stress response genes in wheat from the literature.

Cis elements	Conserved cis motif sequence	References
*ABRE*	ACGTG/ACGT	[27]
*DRE*	CCGAC/RYCGAC	[27], [28]
*MYC*	CANNTG	[29]
*MYB*	CAACNA/CAACNG/TAACNG	[30]
*WRKY*	TGAC	[31]
*ARR*	NGATT	[32]
*DOF*	AAAG	[33]
*RAV*	CAACA	[34]
*GT* − 1	GRWAAW	[35]
*CURECORECR*	GTAC	[36]
*NAC*	CATGTG	[37]

## Discussion

Common bread wheat has one of the most complex genomes known, with six copies of each chromosome, enormous numbers of near-identical sequences scattered throughout, and an overall size of about 17 billion bases. Hence, the development of drought and salt-tolerant varieties has been an important challenge for wheat breeding programs in the recent past [38, 39]. Here, we tried to share in discovering the wheat genome structure by predicting new genes in the huge, recently-released wheat genome. An important genomic approach to identifying drought and salt-stress related genes is based on ESTs generated from different cDNA libraries representing stress-treated tissues collected at various stages of development. The clustering of EST sequences generated from abiotic stress-treated cDNA libraries provides a primary vision of the activated genes involved in stress responses. First, we had an overview for all EST-contigs, then expected new genes with their structures, locations on the genome, and functions. We know there are several classes of TFs involved in the activation of stress response genes in plants. In our study, 14 TF families were identified, of which MYB-HB-like was most abundant, followed by AUX-IAA, WD40-like, AP2-EREBP, C2H2, and bZIP. There are a lot of studies showing the importance of these TF families in abiotic stress tolerance. In Arabidopsis, overexpression of OsMYB3R-2 increases tolerance to cold, drought, and salt stress [40]. Moreover, overexpression of OsMYB48-1 in rice improved tolerance to drought and salinity stresses [41]. In response to abiotic stress, transcription of genes IAA5 and IAA19 are directly promoted by means of several DREB/CBF TFs; furthermore, tolerance to stress decreases with recessive mutations in these IAA genes, providing a role of auxin in abiotic stress [42]. Evidence showed that TaWD40D was associated with tolerance to abiotic stresses in wheat [43]. Relevant to AP2-EREBP, the constitutive expression of OsEREBP1 in rice enhances survival under abiotic or biotic stress conditions [44]. Other TFs, such as C2H2, have been found to play a substantial role in biological processes and response to various abiotic stresses, including oxidative, salt, cold, and drought stresses in rice [45, 46]. This suggests that the TF gene of C2H2-ZFPs (a type of zinc finger protein transcription factor with cysteine (C) residues and histidine (H) residues) are likely related to stress responses and multiple physiological processes in the drought and salt tolerance of wheat also [47]. Additionally, overexpression of GhABF2, encoding bZIP TF in cotton, significantly improved both drought and salt-stress tolerance in *Arabidopsis*[48]. The enzymes, were classified into three groups, transferases, oxidoreductases, and hydrolases with 30 (35.29%), 22 (25.88%), and 14 (16.47%), respectively. These results agree with another study investigating drought and salinity EST in chickpea [49]. We acquired the putative functions of EST-contigs using BLASTX against the protein databases and organized the sequences into three main GO categories. In this study, some stress signaling pathways were represented like, “oxidative phosphorylation”, “calcium signaling pathway”, “flavonoid bio- synthesis”, “fatty acid biosynthesis” and “biosynthesis of other secondary metabolites” (S3 Table). At whole transcriptome level, KEGG pathway is useful techniques for prediction genes and their functions. In Addition, These types of analyses provide a good picture of sequence functions that are effective in specific stresses. A computational approach was performed to align sequences to the fully annotated assembly of hexaploid wheat, IWGSC RefSeqv1.0, using BLAT. Additionally, in-silico functional annotation was carried out using BLASTX and the InterPro database for the 15 EST-contigs considered as candidate drought- and salt-responsive genes (Tables 2 and 4). Three positions were detected for contig54 and contig71 on the three subgenomes A, B, and D. Functional annotation showed that contig54 had similarity with RWP-RK TFs, which have a function associated with abiotic stress in rice [50]. However, contig71 was comparable in function to chaperone DnaJ 10-like, also known as Hsp40 (40-kDa heat hock protein). A study in *Arabidopsis* showed that overexpression of DnaJ (Hsp40) is shared in salinity stress tolerance [51]. The Hsp40 is a co-chaperone to Hsp70 chaperones and together they aid the refolding of non-native proteins under both normal and stress conditions and make up a set of distinguished cellular machines that help with a broad range of protein-folding processes in almost all cellular compartments [52, 53]. Moreover, Hsp40 is involved with abiotic stress-responsive genes [54].

**Table 4:** List of candidate genes related to drought and salinity stresses.

Nam. Contig	Gene Putative Function
*contig*54	RWP-RK transcriptio
*contig*71	Chaperone dnaJ 10-like
*contig*85	REF SRPP-01784
*contig*138	Unknown function wound-induced
*contig*173	Nodulin-related 1
*contig*222	Polygalacturonase inhibitor
*contig*232	Cation transport chaC
*contig*239	Uncharacterized
*contig*274	Early responsive to dehydration 15-like
*contig*278	Early responsive to dehydration 15-like
*contig*293	Serine/arginine-rich proteins
*contig*319	Jacalin-related lectin 9-like
*contig*320	Predicted protein
*contig*325	Ankyrin repeat domain-containing 2A
*contig*347	Transcription factor MYB59-like

Two loci in subgenomes A and D for contig173 and contig293 were separately identified. Functionally, contig173 was analogous to nodulin-related 1 which is involved in heat stress response [55]. However, contig293 appeared to correspond to serine/arginine-rich (SR) proteins; alternative splicing of SR pre-mRNAs is altered by various stresses, increasing the probability of the fast reprogramming of the entire transcriptome by these major regulators of splicing external signals [56]. Although contig278 had two locations on subgenomes B and D, and contig274 had one location on A, the annotation for both sequences is the same and similar to the function of early responsive to dehydration (ERD) genes. These are defined as genes that are rapidly activated during drought stress, and early responsive to dehydration 15 is a new transcription factor and integrates stress signaling pathways [57, 58]. Subgenome A also had locations for contig222, contig325, and contig320. Functional homology for contig222 was detected as polygalacturonase inhibitor and has been observed as being significant for common resistance to biotic and abiotic stresses and also in the mechanisms for cell wall repair [59, 60]. The contig325 was similar to Ankyrin repeat domain-containing 2A, which is involved in regulation of hydrogen peroxide levels during abiotic stress [61].

Despite contig320 being on subgenome A and contig239 on subgenome B, both were similar to uncharacterized predicted proteins. Many discovered proteins have currently unknown roles or putative functions, possibly because of hexaploid wheat’s genome nature, huge size, and complexity. According to our study, these genes are thought to be involved in tolerance to drought and salinity stress. Positions for contig85, contig138, contig232, contig319, and contig347 were identified on subgenome D. Among these, contig85 showed similarity with rubber elongation factor (REF) and small rubber particle protein (SRPP), which are part of a larger plant stress-related protein family that respond to hormones and abiotic stresses [62, 63]. In addition, contig138’s function was similar to wound-induced proteins, which are activated in response to plant wounding. The wounding can be biotic (such as from infections and damage from herbivores) or abiotic (mechanical). Water stress is one of the vital mechanisms of the wound response in tomato [64] and carrot [65]. In a study on rice gene OsCTP similar to E.coli cation transport protein and may be related to general defense against different environmental stresses [66], similar to Contig232 of the present study. Also, in another study on rice, they authors found up-regulation for cation transporter genes after salt stress [67]. Contig319 showed similarity to jacalin-related lectin 9-like in that its expression in wheat was tissue-specific and mostly inducible by abiotic and biotic stresses and stress hormones [68]. The TF MYB59 regulates the expression of several genes and is known to respond to abiotic stress [69]. In a study on Arabidopsis, the authors found that the overexpression of AtMYB44 enhances stomatal closure in transgenic that leads to increase abiotic stress tolerance [70] and contig347 showed homology with this protein. Additionally, another in-silico analysis was used to validate the candidate genes. This was achieved by examining their cis-regulatory elements in the promoter region of the predicted genes using a set of cis-regulatory elements, related to both of the studied traits, calculated from different resources [27]. The majority of these cis-elements were located in the candidate genes. Previous studies showed the importance of cis-regulatory elements in stress adaptation in plants. Therefore, the presence of these cis-elements in the promoter region of candidate genes indicates their possible involvement in drought and salt-stress response mechanisms in *T. aestivum*[71].

## Conclusion

Wheat is the most agriculturally important crop in the world and is severely affected by drought and salinity. Advanced biotechnological methods need to be applied to develop drought- and salt-tolerant wheat varieties. This study focused on providing in-silico analyzed common drought- and salt-responsive genes in wheat. In addition, identification of highly expressed genes under drought and salinity conditions has multiple significance: (i) It provides a more comprehensive understanding of the transcriptional responses to drought and salinity stresses. (ii) It helps identify the role of individual genes in stress responses. (iii) It assists in identifying stress-responsive promoters and the responsible cis-elements within them. (iv) It identifies the cross- tolerant genes induced by both drought and salt stresses. We reported 22 putative drought- and salinity-related genes in hexaploid wheat that have different functions, and some of them are accumulated or directly upregulated after drought and salt stresses (like contig71, contig274, contig278, contig232, contig319, and contig347). The genes can be better validated later by designing new primers to be tested and undertaking researches for unknown function predicted genes to be used in wheat breeding programs.

## Supporting information

S1 Table Transcription factors in the EST-contigs. (XLSX)

S2 Table Enzymes found in EST-contigs and their pathways. (XLSX)

S3 Table KEGG classification of T. aestivum contigs. (XLSX)

S4 Table Coding positions of the in-silico validated candidate genes with TSS PolyA and CDs between them. (XLSX)

S5 Table Presence of salt and drought stresses cis-element in the candidate genes. (XLSX)
